# Chloroplast DNA Structural Variation, Phylogeny, and Age of Divergence among Diploid Cotton Species

**DOI:** 10.1371/journal.pone.0157183

**Published:** 2016-06-16

**Authors:** Zhiwen Chen, Kun Feng, Corrinne E. Grover, Pengbo Li, Fang Liu, Yumei Wang, Qin Xu, Mingzhao Shang, Zhongli Zhou, Xiaoyan Cai, Xingxing Wang, Jonathan F. Wendel, Kunbo Wang, Jinping Hua

**Affiliations:** 1 Department of Plant Genetics and Breeding/Key Laboratory of Crop Heterosis and Utilization of Ministry of Education/Beijing Key Laboratory of Crop Genetic Improvement, China Agricultural University, Beijing 100193, China; 2 State Key Laboratory of Cotton Biology, Institute of Cotton Research, Chinese Academy of Agricultural Sciences, Anyang 455000, Henan, China; 3 Department of Ecology, Evolution and Organismal Biology, Iowa State University, Ames, IA 50011, United States of America; Saint Mary's University, CANADA

## Abstract

The cotton genus (*Gossypium spp*.) contains 8 monophyletic diploid genome groups (A, B, C, D, E, F, G, K) and a single allotetraploid clade (AD). To gain insight into the phylogeny of *Gossypium* and molecular evolution of the chloroplast genome in this group, we performed a comparative analysis of 19 *Gossypium* chloroplast genomes, six reported here for the first time. Nucleotide distance in non-coding regions was about three times that of coding regions. As expected, distances were smaller within than among genome groups. Phylogenetic topologies based on nucleotide and indel data support for the resolution of the 8 genome groups into 6 clades. Phylogenetic analysis of indel distribution among the 19 genomes demonstrates contrasting evolutionary dynamics in different clades, with a parallel genome downsizing in two genome groups and a biased accumulation of insertions in the clade containing the cultivated cottons leading to large (for *Gossypium*) chloroplast genomes. Divergence time estimates derived from the cpDNA sequence suggest that the major diploid clades had diverged approximately 10 to 11 million years ago. The complete nucleotide sequences of 6 cpDNA genomes are provided, offering a resource for cytonuclear studies in *Gossypium*.

## Introduction

Cotton is the most important fiber crop plant in the world. Four species were domesticated and remain under cultivation today, the New World allopolyploids *G*. *hirsutum* and *G*. *barbadense* (2n = 52), and the Old World diploids *G*. *arboreum* and *G*. *herbaceum* (2n = 26) [1–2]. The primary cultivated species is Upland cotton (*G*. *hirsutum* L.), which accounts for more than 90% of global cotton fiber output. *Gossypium* includes 52 species, including 6 allotetraploid species and 46 diploids [2]. The nascent allopolyploid spread throughout the American tropics and subtropics, diverging into at least six species, namely, *G*. *hirsutum* L. (AD_1_), *G*. *barbadense* L. (AD_2_), *G*. *tomentosum* Nuttalex Seemann (AD_3_), *G*. *mustelinum* Miersex Watt (AD_4_), *G*. *Darwinii* Watt (AD_5_), and *G*. *ekmanianum* (AD_6_) [1–2]. The diploid *Gossypium* species have been shown to comprise 8 monophyletic genome groups, A, B, C, D, E, F, G and K group [1,3–4].

Because of its economic importance and its value as a model for evolutionary studies, there is a rich history of molecular phylogenetic work in *Gossypium* (reviewed in [1–2]). These studies, although based mostly on a set of nuclear genes [5], or chloroplast DNA restriction sites [6], indicate low levels of divergence among species and even clades, and suggest a rapid, early diversification of the primary cotton lineages, such that many of the branch resolutions remain in question. Divergence among diploid clades was estimated to have occurred rapidly following an initial split around 6.8 MYA [5,7].

With the advent and rapid development of next-generation sequencing technologies [8–10], cotton genomics research has progressed rapidly in the last several years, such that nuclear genome sequences have now been published for model diploids D-genome [11–12], A-genome [13] and for the allopolyploids *G*. *hirsutum* [14–15], *G*. *barbadense* [16–17]. In addition, a large number of organelle genome sequences have been published [18–22]. Chloroplast DNA sequences have long been a major data source for plant phylogenetic inference [23–25], with both the relatively conserved coding and more highly diverged non-coding regions being useful at different levels [25–26]. Because of its abundance and relatively uniform size and organization [18–20,27], complete chloroplast (cp) genome sequences from *Gossypium* should be readily alignable and hence useful for phylogenetic analysis. As an initial step in this direction, Xu et al., [20] used complete nucleotide sequences of 12 cp genomes from four diploids and eight tetraploids to analyze the origin and evolution of allotetraploids.

To provide insight into divergence, phylogenetic relationships and cp genome structural variation across the entire genus, we performed a comparative analysis of 19 (13 unpublished) *Gossypium* cp genomes (2 from tetraploid species and 17 from diploids), including those from 6 diploids not previously sequenced. Phylogenetic analyses were performed using both nucleotide and indel data. Our comparative analyses of these 19 genomes provided detailed information on divergence within and between clades, including the age of divergence among species.

## Materials and Methods

### Plant materials and chloroplast isolation

Fresh leaves from six species representing four genome groups in *Gossypium* were collected for chloroplast extraction and sequencing. All materials were obtained from the National Wild Cotton Nursery, in Sanya, China, which were issued the permission by the authority: Cotton Research Institute, Chinese Academy of Agricultural Sciences, Anyang, Henan, China. Chloroplast DNA was prepared following a previous published protocol [20,28]. Illumina libraries with paired-end, 90bp read, were generated using Illumina sequencing method on HiSeq2000 at Beijing Genomics Institute (BGI).

### Chloroplast assembly and annotation

Raw reads were filtered using Bowtie2 [29] for possible nuclear and/or mitochondrial contamination by extracting only those reads that showed similarity with the published *G*. *hirsutum* (AD_1_) cp genome sequence. Chloroplast reads were subsequently assembled using a combination of Phrap [30] and Velvet [31] (hash length = 21, cov_cutoff = 30). Each inverted repeat (IR) region was specifically targeted using two long PCR reaction (each producing ~13 kb fragments), whose products were purified for sequencing separately with Illumina. Chloroplast genes were annotated using an online DOGMA tool [32] using *G*. *hirsutum* (AD_1_) as a reference sequence. The sequences of identified tRNA genes were obtained using both DOGMA and tRNAscan-SE [33]. Genome maps were drawn with OGDRAW [34].

### Estimation of evolutionary divergence between sequences

The whole genome sequences were aligned with genome specific aligner: Alignathon [35]. Sequence alignments for each coding, intronic, and intergenic spacer regions were carried out by different alignment methods combining CLUSTALW [36], MUSCLE [37] and MAFFT [38] to address the alignment reliability, which demonstrate that using different alignment methods does not change the main results. The number of indels and substitutions were calculated by a custom Perl script. P-distances for any two genomes, genes, or non-coding regions were calculated with MEGA5.05 [39].

### Phylogenetic analyses and divergence time of *Gossypium* diploid clades

The most closely related and publicly available chloroplast sequence was determined via BLAST [40] against publicly available databases using *Gossypium hirsutum* as the query (outgroup = *Theobroma cacao*, Malvales, GI:342240206). Initially, a DNA substitution model for our data sets was selected using jModelTest version 2.1.4 [41] and the Akaike Information Criterion (AIC). Among the 88 models tested, the general time reversible (GTR) including rate variation among sites (+ G) and invariable sites (+ I) (= GTR + G + I) model was chosen as the best fit to our data sets, followed by the Transversional model + G + I and GTR + I models. Maximum likelihood (ML) trees were generated for all phylogenetic comparisons using either in MEGA5.05 [39], PhyML 3.0 [42] or RAxML [43], all using a General Time Reversible (GTR) model and a rate of Gamma distributed with invariant site (G+I) Bootstrap support (BS) values for individual clades were calculated by running 1,000 bootstrap replicates of the data. Gaps/missing data were evaluated both as complete deletions and as missing data, both of which gave the same topology in each case. Bayesian analysis of the ML trees was conducted by MrBayes [44] under GTR gamma with the following parameters: 3 runs with four chains for 10 million generations and using a burn-in fraction of 25%.

To evaluate phylogenetic signal present in the indel data, we coded gaps using modified complex coding [45] as implemented in SeqState [46]. The indel data was evaluated both separately and in conjunction with the substitution data using RAxML [43]. Again, a GTR model was invoked for the nucleotide substitution partition, while the MULTICAT model (as implemented in RAxML) was invoked for both the standalone and state-data partition of the combined analysis, and both trees were generated using 1000 alternative runs on distinct starting trees and rapid bootstrapping with consensus.

Divergence time was estimated for the 78 concatenated chloroplast protein-coding exons dataset using PhyloBayes 3.3f [47], using the autocorrelated Lognormal relaxed-clock mode [48] and the tree generated from the above dataset and CAT+GTR model. For the molecular clock analysis, a birth-death prior on divergence time and fossil calibrations with soft bounds were used, and we selected three fossil calibrations for *Gossypium* vs *Theobroma*, ancestors shared between A and D subgenomes and the split of A and AD genomes (S8 Table). The range of fossil age was collected from relevant literature on fossils [49] and a recent molecular calculation of the *Gossypium* clades [50–51]. We allocated 10% of the probability mass to lie outside each calibration interval. All calculations were performed by running 10,000 generations and sampled every 25 generations (after burn-in of 2,500 generations).

## Results and Discussion

### Size, content and structure of six new *Gossypium* chloroplast genomes

**Fig 1:**
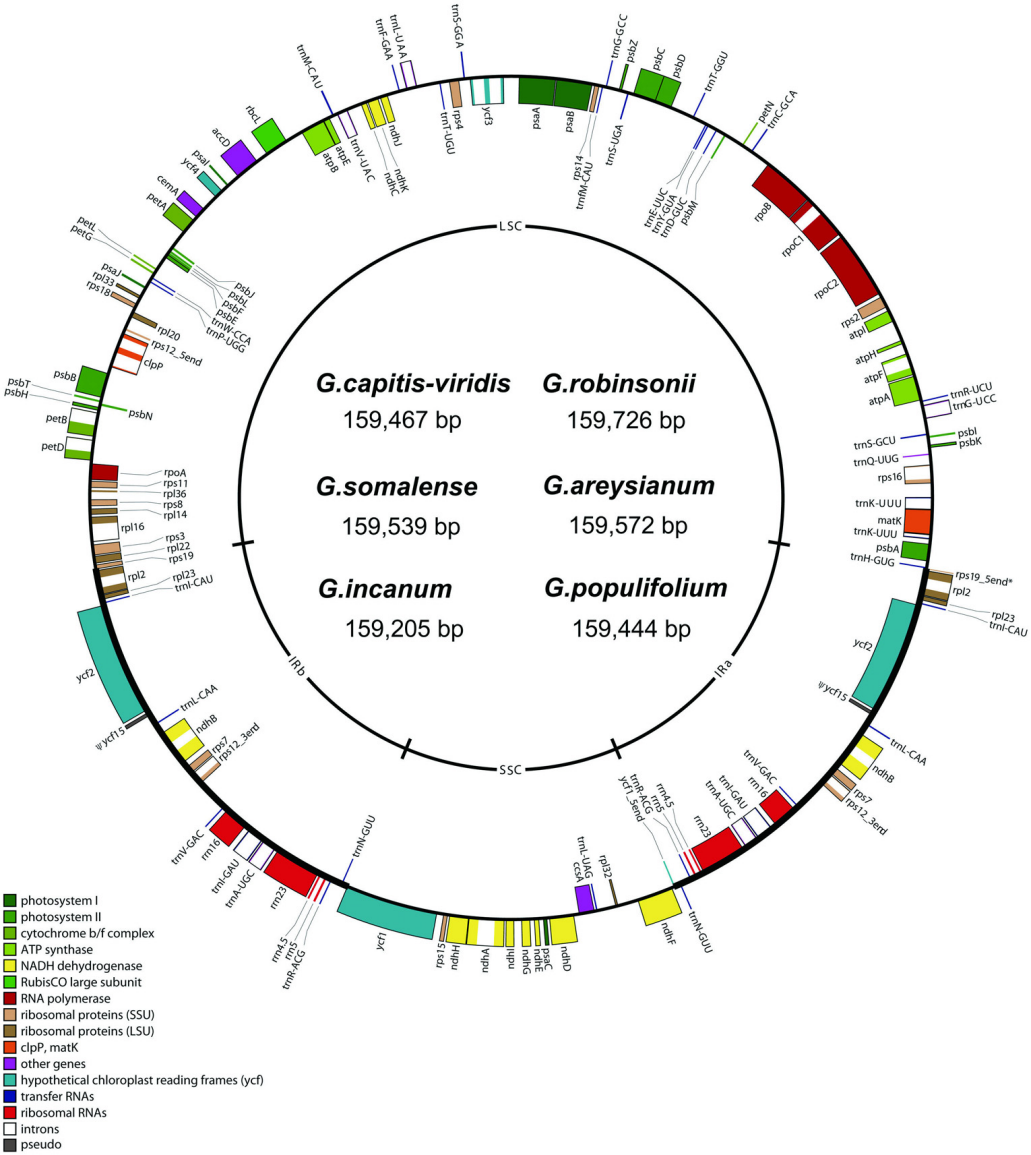
A consensus map of six newly sequenced *Gossypium* chloroplast genomes. Genes on the outside of the outer circle are transcribed in the clockwise direction and genes on the inside of the outer circle are transcribed in the counterclockwise direction. The inner circle delineates the inverted repeat regions (IRa and IRb), the small single-copy region (SSC), and the large single-copy region (LSC). Functional categories of genes are color-coded.

*Gossypium* chloroplast (cp) genomes from six diploid species were newly sequenced for this study, representing four of the eight cotton diploid genome groups (*G*. *robinsonii* C_2_, *G*. *incanum* E_4_, *G*. *somalense* E_2_, *G*. *capitis-viridis* B_3_, *G*. *areysianum* E_3_, *G*. *populifolium* K; GenBank accessions JN019791 to JN019795 and KP221924, respectively). These cp genomes (Table 1) show high identity and similarity in gene content and genome organization with each other and with previously published cotton cp genomes [20], with only minor differences in genome size and composition. The length of these six genomes range in size by only 521 bp, from the largest (*G*. *robinsonii*, C_2_, 159,726 bp) to the smallest (*G*. *incanum*, E_4_, 159,205 bp), with most of the size differences occurring in the large single-copy (LSC) region (Table 1 and Fig 1). Notably, all are smaller than the previously published *G*. *hirsutum* cp genome [18] by more than 500 bp. All six cp genomes contain 112 genes, including 78 protein-coding genes, 4 ribosomal RNA genes and 30 tRNA genes, and 17 duplicated genes located in IR region (Fig 1, S2 Table). Both the length of the coding regions and the overall GC content vary minimally as well (<1% each; Table 1).

**Table 1:** General features of six *Gossypium* chloroplast genomes.

Species	Genome	Total Size (bp)	LSC Size (bp)	IR Size (bp)	SSC Size (bp)	G+C (%)	Coding ratio (%)	GenBank accessions
*G*. *capitis-viridis*	B_3_	159,467(-834)	88,065 (-752)	25,602(0)	20,198(-82)	37.32	56.77	JN019794
*G*. *robinsonii*	C_2_	159,726(-575)	88,359(-458)	25,582(-20)	20,203(-77)	37.17	56.70	JN019791
*G*. *somalense*	E_2_	159,539(-762)	88,150(-667)	25,569(-33)	20,251(-29)	37.37	56.76	JN019793
*G*. *areysianum*	E_3_	159,572(-729)	88,182(-635)	25,569(-33)	20,252(-28)	37.37	56.75	JN019795
*G*. *incanum*	E_4_	159,205(-1096)	87,879(-938)	25,565(-37)	20,196(-84)	37.39	56.87	JN019792
*G*. *populifolium*	K	159,444(-857)	88,197(-620)	25,577(-25)	20,093(-187)	37.20	56.97	KP221924

Note: LSC = large single copy region; IR = inverted repeat regions; SSC = small single copy region. The numbers in parentheses indicate the size comparison of that region to the corresponding region in the published *G*. *hirsutum* cp genome [16]. As the IR regions are identical, and therefore impossible to distinguish, the IR regions for each chloroplast were assembled as a single repeat.

### Nucleotide divergence among cp genomes of 19 *Gossypium* species

In addition to the six newly presented cp genomes, we also analyzed 13 previously sequenced cp genomes, including representatives of the A, B, C, D, E, F and G genome groups (S1 Table). Not surprisingly, the lowest levels of nucleotide divergence among these 19 species were detected within genome groups, some of which show remarkable uniformity. Within the E-genome, for example, the comparison of *G*. *somalense* (E_2_) and *G*. *areysianum* (E_3_) yielded only a single-nucleotide change in a protein-coding exon and a total of 10 nucleotide substitutions across all non-coding regions, a nucleotide distance of 0.000075; the distance within the A-genome was similarly low (0.000074; S3 Table). Low levels of divergence may not be uniform across genome group, however. For example, the distance between *G*. *incanum* (E_4_) and *G*. *stocksii* (E_1_) (0.000668) was about 8-fold higher than that of *G*. *somalense* (E_2_) and *G*. *areysianum* (E_3_) (S3 Table), making it larger than that found within the B-genome, 0.000284 for *G*. *anomalum* (B_1_) and *G*. *capitis-viridis* (B_3_) and smaller than D-genome, 0.001283 for *G*. *raimondii* (D_5_) and *G*. *gossypioides* (D_6_) that was, lower than for the other two comparisons, as expected based on previous cpDNA analyses [6]. All intra-genomic comparisons are performed. Interestingly, among the Australian cottons, *G*. *sturtianum* (C_1_) was more similar to *G*. *bickii* (G_1_) than to *G*. *robinsonii* (C_2_) and *G*. *populifolium* (K), supporting the proposal [52–53] that *G*. *bickii* has an introgressive ancestry with a maternal donor from the *G*. *sturtianum* lineage. As expected, the divergence among genome groups was typically an order of magnitude larger, ranging from 0.003593 to 0.009612. The pairwise comparisons within A+AD, B, *G*. *sturtianum* vs *G*. *bickii* (C_1_ vs G_1_), D and E groups showed the divergence values less than 0.26% because of their highly close relationship during evolution. In addition, the distances, ranging from 0.26% to 0.53%, contains pairwise comparisons between close *Gossypium* groups, for example, distances between A+AD and F groups, and some comparisons within C + G + K groups (*G*. *populifolium* vs *G*. *robinsonii*, *G*. *populifolium* vs *G*. *bickii*). However, the distances more than 0.53% contained species compared that own a really distant relationship and come from different phylogenetic groups, such as the largest pairwise comparisons distances between C + G + K groups and other five groups. Interestingly, and consistent with the first published phylogenetic data using *Gossypium* chloroplast genomes nearly a quarter of a century ago [6], the species *G*. *robinsonii* (C_2_) shows greater distances to other genome groups than do those of other species.

When diversity is partitioned into coding and non-coding fractions, the non-coding fraction typically displayed two to three times the variability of the coding regions (S4 Table). Some comparisons, but only when divergence amounts are very low, show the opposite pattern; between *G*. *herbaceum* (A_1_) and *G*. *africanum* (A_1-a_), for example, the nucleotide distance (total, including both non-synonymous and synonymous substitutions) was 0.000383 in coding regions while 0.000222 for non-coding regions (S4 Table). The 78 protein-coding exons had an average distance of 0.003109, ranging from no substitutions in 8 genes to a distance of 0.010599 in *ycf1* averaged for all pairwise comparisons among the 19 genomes. The eight completely conserved genes (S5 Table) were *petL*, *psbE*, *psbH*, *psbL*, *psbM*, *psbN*, *psbT* and *rpl23*, of which six (*psbE*, *psbH*, *psbL*, *psbM*, *psbN* and *psbT*) belong to the Photosystem II functional category (15 genes in total), potentially indicative of intense selective constraint. We also analyzed the nucleotide divergence among 8 species of *Oryza* (data not shown), and found six completely conserved Photosystem II genes (*psbE*, *psbI psbL*, *psbM*, *psbN*, *psbT*), 5 of which are shared with *Gossypium*. These results support the conclusion that these genes evolve under intense purifying selection.

Non-coding chloroplast regions in *Gossypium* comprise 112 intergenic spacers (excluding one IR region) and 19 introns, 17 of which were identical in sequence among all nineteen *Gossypium* species: the spacers *psbD*/*psbC*, *psaB*/*psaA*, *atpE*/*atpB*, *psbL*/*psbF*, *psbF*/*psbE*, *psbN*/*psbH*, *rps3*/*rpl22*, *rpl2*/*rpl23*, *trnI*-*CAU*/*ycf2*, *ndhB*/intron, *rps7*/*rps12*_3end, *trnV*-*GAC*/*rrn16*, *trnI*-*GAU*/*trnA*-*UGC*, *trnA*-*UGC* intron, *trnA-UGC*/*rrn23*, rrn23/rrn4.5 and *ndhH*/*ndhA* (S5 Table). These highly conserved intergenic regions may indicate co-transcription or a conserved regulatory role for these spacers. Overall, the average nucleotide distance for the non-coding cp regions was 0.010798, or as noted above, 3.4 times larger than was observed for coding regions.

### Chloroplast genome phylogeny of *Gossypium* is congruent with the chloroplast gene-based phylogeny

**Fig 2:**
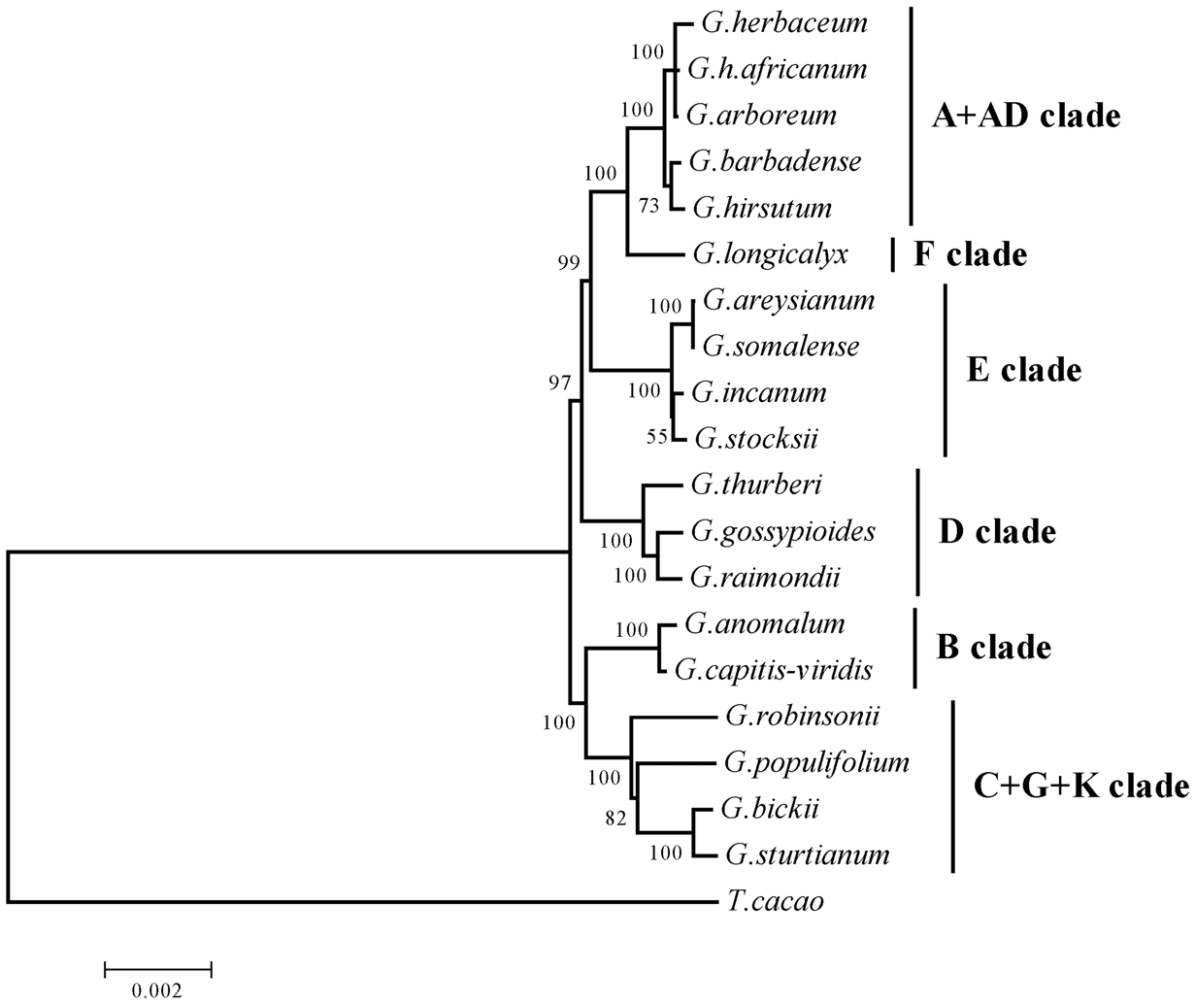
Maximum likelihood (ML) phylogenetic tree of 19 *Gossypium* species based on several analyses, including whole genome sequences, 78 concatenated chloroplast protein-coding exons sequences and indel-coded data. *Theobroma cacao* was used as outgroup. Bootstrap values for all major divergences were high (>90%) on the corresponding nodes (Bayesian tree is similar, and therefore not displayed).

The phylogeny of *Gossypium* has been previously evaluated [5] using limited plastid and nuclear data. In the most recent analysis using both chloroplast and nuclear data, inconsistencies in the basal branching patterns of the genus were both observed and well-supported. Statistical analyses of incongruence provided greater support for the nuclear tree topology [5], as opposed to the cp-resolved topology. To revisit this inconsistency, we inferred phylogenetic relationships among the eight *Gossypium* genome groups using a concatenated analysis of all 78 chloroplast protein-coding genes and *Theobroma cacao* as an outgroup. The topology of the resulting tree (Fig 2) was congruent with that previously reported [5], which evaluated only four cp loci, two genes and two non-coding regions. To explore this further, we performed both a separate analysis for each of the 78 genes as well as an analysis of the molecule as a whole. Only one individual gene, *ndhF*, showed the same topology as the concatenated analysis, an unsurprising result given the low amount if divergence within each gene and hence the lack of resolution for many gene-clade combinations. When the entire cp genome was considered (gaps excluded), support for the topology increased, with a minor discrepancy in the placement of *G*. *populifolium* (K genome; S1 Fig). These observations are perhaps unsurprising, as the cp genome as a whole is subject to the same evolutionary influences as its smaller components (unlike the nuclear genome), yet it is notable that the results from the analysis of the entire genome are consistent with those previously reported for few loci (if better supported), which suggests that, at the phylogenetic level evaluated here, a small fraction of the chloroplast can adequately serve to represent the evolutionary history of the whole [5].

The resolution of intraclade relationships, however, was largely reliant on the substantial sequence information afforded by whole cp genome sequencing. Interestingly enough, the phylogenetic analyses conducted here indicate that this may be true for some intraclade relationships, which were far less distinct than others in the same genome group. In the E-genome, for example, of the four species evaluated, two (*G*. *somalense* and *G*. *areysianum*) were nearly identical in their chloroplast genomes, whereas the other two E-genome species (*G*. *stocksii* and *G*. *incanum*) species in E clade here had more distinct sequences. This high similarity was also present for *G*. *africanum* (A_1-a_) and *G*. *arboreum* (A_2_), which, as previously noted [4,20,54] are distinguishable morphologically, yet may still be in the initial stages of species differentiation (as indicated by the low level of sequence divergence). This indicates that, while limited sampling of the chloroplast molecule may be sufficient for interclade phylogenetics, more extensive sampling is required for adequate resolution at close specific relationships.

### Structural variation among cotton chloroplast genomes

Insertion-deletion polymorphisms (indels) may be another useful source of phylogenetically informative characters [55–57]. Phylogenetic analysis of indel patterns has been broadly applied, from discerning interfamilial relationships among mammals [58], to reconstructing generic level plant phylogenies [56], to species recognition issues in *Gossypium* [59]. The most recent phylogenetic analysis of relationships among diploid cotton genome groups [5], also used indel polymorphisms as a line of evidence; however, this dataset was restricted to few indels derived from both the nuclear and chloroplast genomes, in roughly equal proportions. To revisit this issue, we scored and evaluated the pattern for 1420 indels in the 19 *Gossypium* and *T*. *cacao* cpDNA protein-coding and non protein-coding regions (S6 Table).

#### IR junction polymorphisms are present, yet phylogenetically uninformative

Although the cp genomes studied here are extremely similar in structure, size, gene number and gene order, numerous small indels differentiate the genomes even among closely related species. Of the 1420 indels that differentiate these cp genomes, 69 (5 in coding and 64 in non-coding regions) are located in the IR region (S6 Table). Given that the IR region is the only place in the cp genome that recombination is expected, we analyzed the junction between these regions and the single copy regions separately.

**Fig 3:**
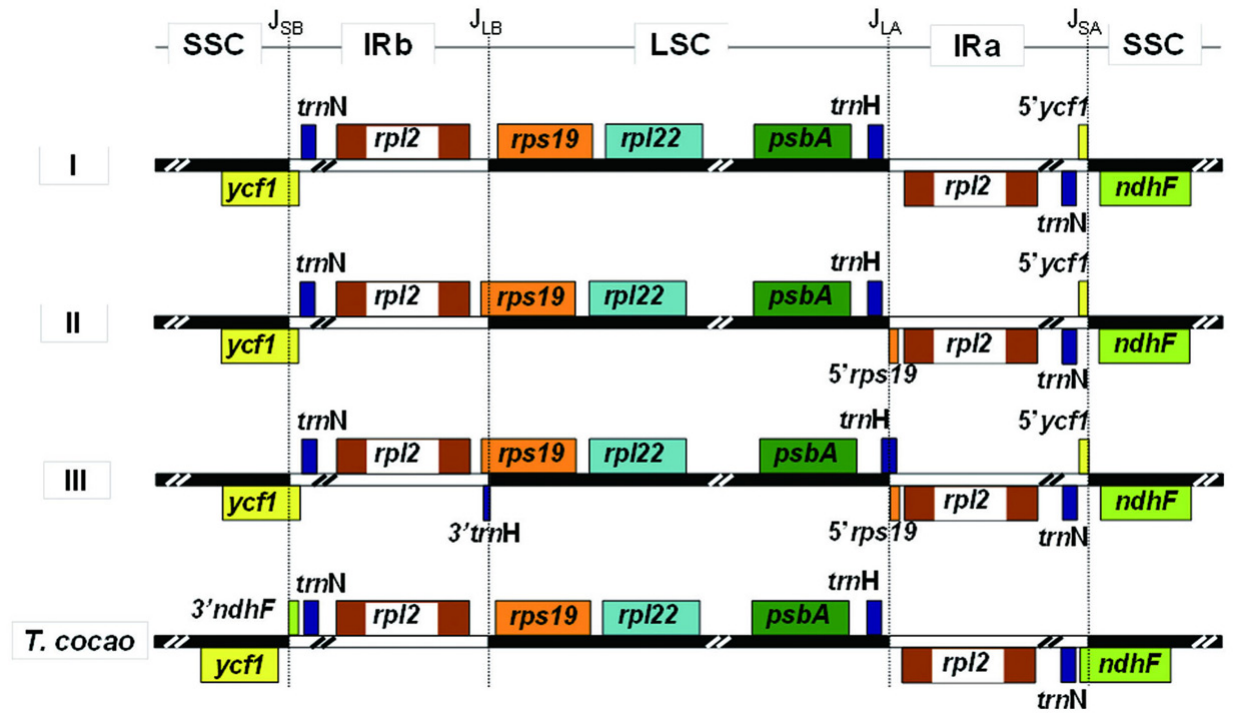
Three types of junction region models for *Gossypium* chloroplast genome. **Type I**, *rps19* and *trnH*, entirely located in LSC region with no any overlap fragments in IR region. **Type II**, *rps19* across the point of J_LB_, part fragment of 5’rps19 located in IRa region, *trnH* perfectly located in LSC region. **Type III**, *rps19* across the point of J_LB_ and *trnH* across the point of J_LA_, part fragment of 5’*rps19* and 3’*trnH* located in IRa and IRb region, respectively. Also see S1 Fig for phylogenetic placement of each IR junction type.

When analyzed using *Theobroma cacao* as an outgroup, three IR junction types (I, II, and III) were detected (Fig 3), which differ in their placement of *rps19* and *trnH* at the IR-LSC junction site. In assigning an IR junction type (S1 Fig) to each species cp genome, it becomes readily apparent that, while there may be some phylogenetic signal in these IR junction polymorphisms, there must also exist a certain amount of fluidity in their expansions/contraction. For example, of the four E-genome species sampled, three belong to Type II, whereas the other belongs to Type I; the three D-genome species evaluated were likewise split between Types I, II and III. This is indicative of evolutionary fluidity of IR expansion/contraction within genome groups; when plotted against the phylogeny (S1 Fig), it becomes clear that the pattern of IR junction types observed here represents as many as 6 independent switches, independent of whether we invoke a sequential, two-step expansion [60] or if we allow the IR junction types to switch equally among the three. The potentially labile nature of the IR region is further underscored by the observations that: (1) the IR region in cotton has expanded (relative to *T*. *cacao*) to include part of *ycf1*, (2) the *T*. *cacao* IR region has expanded (relative to *Gossypium*) to include part of *ndhF*. Further analyses involving many related species and genera are necessary to understand the evolution of the IR junction.

#### Phylogenetic signal in chloroplast indels supports the chloroplast phylogeny, is incongruent with nuclear data

The utility of indels for phylogenetic purposes has been discussed, leading to the general conclusion that indel polymorphisms can be informative characters with low levels of homoplasy [57], often supporting or refining the inferences determined through substitution data [55–58]. The use of indel data for the most recent analysis of interclade relationships in *Gossypium* [5], however, presented a different scenario. That is, while the chloroplast loci evaluated in that study resolved relationships that were also resolved here (Fig 2), the indel data presented there (Cronn 2002, Fig 4C) suggests an entirely different relationship among genome groups where the D-genome represents the basal-most branchpoint and the African B-genome is more closely related to the F-A genome clade than to the Australian species. This latter phylogeny has been the most widely accepted [2,4], in part due to the statistical analysis [5]; however, challenges to the branching order have been cited [54].

**Fig 4:**
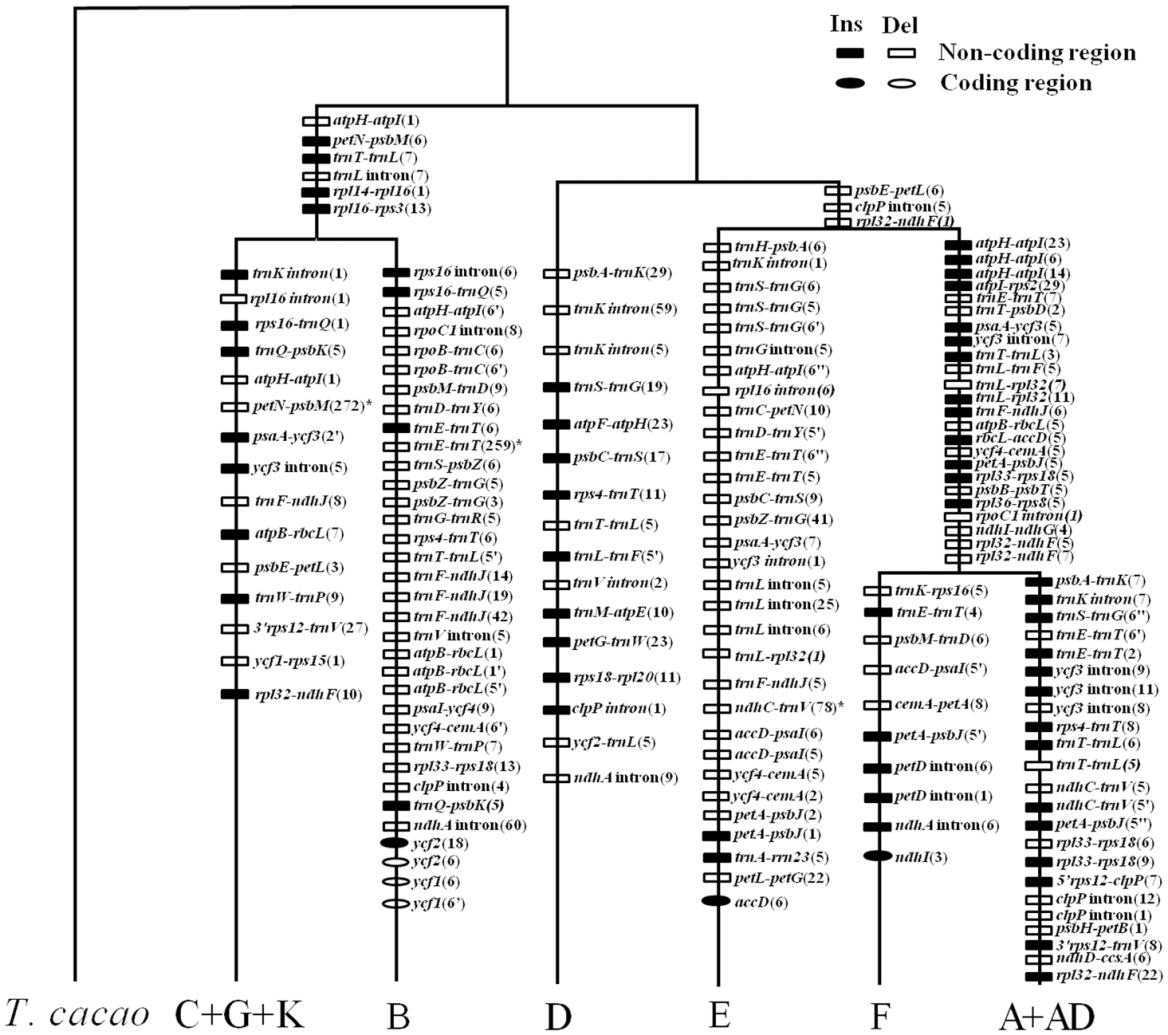
Inferred gains and losses of chloroplast genomic features during the evolution of *Gossypium* diploid species. Genomic characters were mapped on the tree. Gains and losses of characters are indicated by solid and hollow symbols, respectively. *: the indels length aligned with *G*. *hirsutum*. The number in parentheses represents the length of indels.

To evaluate possible discrepancies between indel and substitution-derived data, we used maximum likelihood to reconstruct phylogenetic trees using both indel only data, and concatenated indel + substitution information. Again, both the indel-derived data and the indel + substitution data recovered a tree either identical (indel + substitution) or nearly identical (indel only) to that recovered by substitution data alone. This is in constrast to the indel data presented in Cronn et al. [5], but perhaps not surprisingly so. The indel data previously used was a combination of nuclear and chloroplast derived indels, in a roughly 50–50 proportion, with the resulting tree more closely resembling the nuclear gene tree than the chloroplast gene tree. That the nuclear and chloroplast data resolve a different, contrasting tree from the chloroplast indel data alone indicates a possible incongruence between the nuclear and chloroplast genomes of *Gossypium*. This may be partially explained by a hypothesis tentatively put forth by Cronn and Wendel over 10 years ago [53], which discussed the propensity for cotton species to experience cryptic introgressions among diverse species, often over great distances. Although cotton species typically exist as small, isolated populations, the genus has a remarkable tendency for long-distance dispersal and introgression among species that seem unlikely to geographically meet. This propensity for long-distance dispersal and introgression is well-discussed [53]; however, the observations most applicable to the present are those of multiple chloroplast introgressions among species. As mentioned above, the close inter-clade relationship between *G*. *sturtianum* and *G*. *bickii* can be attributed to introgression of a *G*. *sturtianum*-like chloroplast into the *G*. *bickii*, and a similar observation can be made between *G*. *raimondii* and *G*. *gossypioides*. More ancient introgression events can be difficult to readily pinpoint; however, the incongruence between data types (nuclear versus chloroplast), as well as morphological characters atypical of the genome group, suggest an ancient introgression between a B-like ancestor with an ancestor leading to the Australian (CGK) genome groups. Further, extensive nuclear sampling will be required to determine if the incongruence between these datasets supports these interclade introngression events.

#### Phylogenetic placement of indels and implications for genome size

Indel accumulation was primarily restricted to non-coding regions (S7 Table), which contained over 96% of the indels scored (S6 Table). Of the 1,420 indels that differentiate these cp genomes, only 55 occurred in gene regions, with the length of these rare indels typically occuring as a multiple of three (to preserve protein coding capacity). Interestingly, and as observed in other species [61–62], the terminal codon of *rbcL* has undergone considerable variation among the species analyzed. Also notable are the multiple events that occurred in some *ycf* gene family members, which is identical to previous results [63]. Indels in the non-coding regions were far more frequent and variable in size (S6 Table; S7 Table), ranging in length from 1 to 272 bp, with lengths 1, 5, and 6 bp occurring most frequently, an observation consistent with an earlier report [20].

To evaluate the rate of indel formation among related genome groups, we phylogenetically mapped the phylogenetic polarizable insertions and deletions onto the *Gossypium* phylogeny produced here (Fig 4). As is perhaps expected by the types of mutational processes expected in the chloroplast (e.g. slipstrand mispairing), for any given branch, there were typically nearly equivalent numbers of insertions and deletions; however, two notable exceptions exist. In both the B- and E- genome lineages, the number of deletions was greatly increased and greatly outnumbered the insertions (Fig 4). For the B-genome, but not for the E-genome, this created a relative increase in the number of indel events (as compared to sister branches). For the B-genome, there were a total of 34 indels polarized (compared to 15 for the Australian CGK branch), whereas the number of polarized events in the E-genome lineage was similar to that of the lineages leading to F and A+AD (31 in E-genome, versus 34 and 37 in F and A+AD, respectively) (Fig 4).

Genome size evolution itself is a dynamic process involving counterbalancing mechanisms whose actions vary across lineages and over time [7]. While many of these mechanisms are more active and/or restricted to the nuclear and plant mitochondrial genomes, cpDNA intergenic regions are known to often exhibit substantial insertion/deletion (indel) polymorphism within and among plant species [64–67]. This propensity for deletion may, in part, explain the relatively small size of the B- and E-genome chloroplast genomes.

### Divergence times of major clades in *Gossypium*

**Fig 5:**
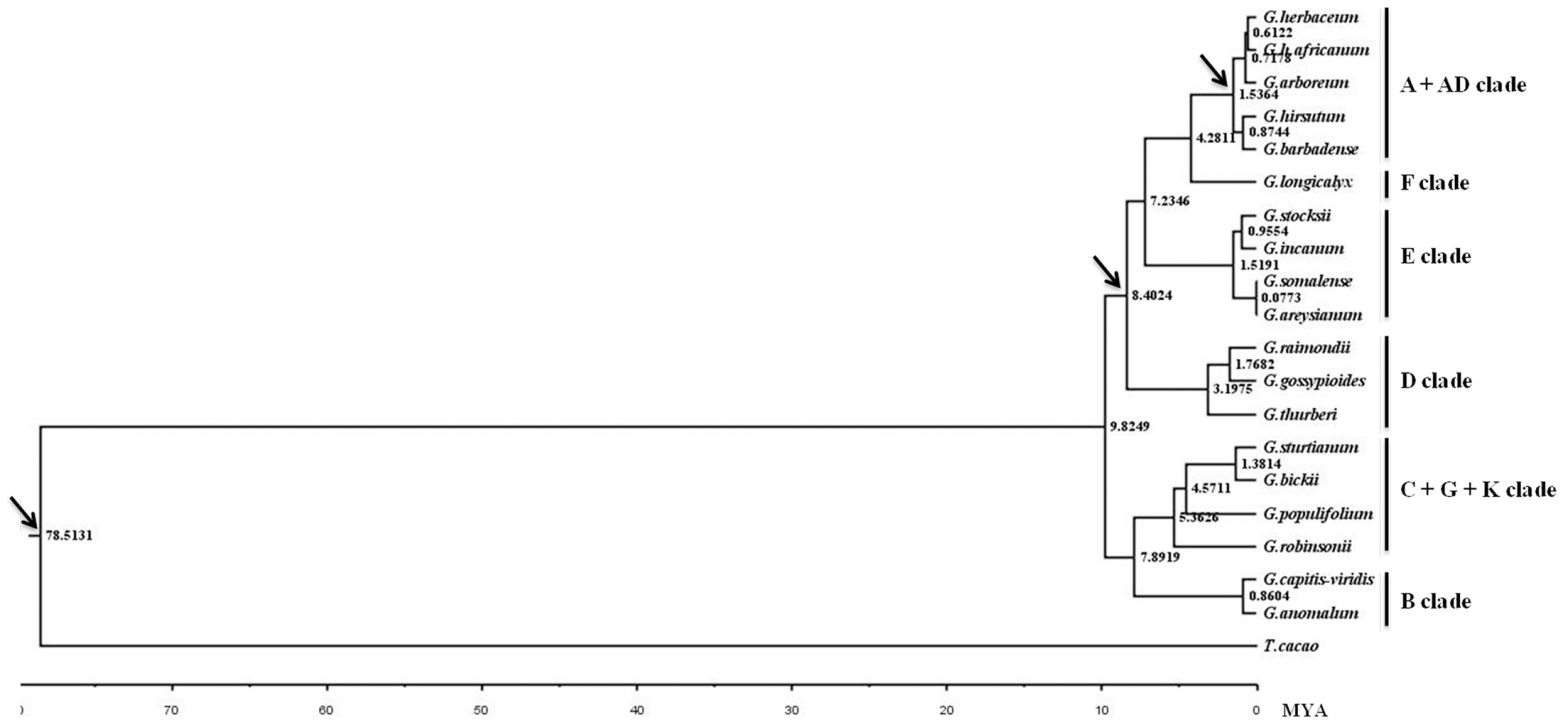
Chronogram showing *Gossypium* phylogeny and divergence time with *T*. *cacao* as an outgroup. Consensus tree presenting divergence dates produces by the PhyloBayes analysis of the 78 concatenated chloroplast protein-coding exons dataset using three fossil calibration points (S8 Table), the autocorrelated Lognormal relaxed-clock mode, the site-heterogeneous mixture CAT+GTR substitution model, and soft bound 10%. A geological time scale is shown at the bottom. The arrows represent for three calibration points.

We used the data gathered here to reevaluate the divergence time for each of the species in this study, using *T*. *cacao* as an outgroup and relaxed molecular clock analyses were performed for our dataset using three calibration points (S8 Table). Prior analyses have put the divergence time for *Theobroma*-*Gossypium* at least 60 million years ago (mya) [49], A-genome diploids native to Africa and Mexican D-genome diploids diverged ~ 5–10 mya [51] and the formation of the allopolyploid at 1–2 mya [50]. The divergence time between each species represented was calculated (Fig 5) with variance around the age estimates (S2 Fig). The divergence time between *Gossypium* and *T*. *cacao* was estimated at ~ 78.5 (56.8–130.8) mya, which is consistent with earlier estimates [49]. While we cannot estimate the formation of the genus itself adequately (without access to a more closely related outgroup), the earliest divergence (between the B+C+G+K-genome clade and the remainder of the genus) was estimated as occurring approximately 9.8 (6.7–13.6) mya, similar to the estimates of the age of the genus [1–2] and consistent with the notion of rapid radiation. Also consistent with prior analyses, which recovered short internodes for most branches, the majority of intraclade divergences fell in the range of 7–9 mya. Interestingly, and perhaps demonstrating yet again the pecularities present in the B-genome, while this clade groups strongly with the Australian clade (C+G+K) phylogenetically, the estimate of divergence time between the B-genome and the remainder of the genus is typically 7.9 (5.0–10.0) mya (Fig 5 and S2 Fig), which is similar to the radiation times calculated for the rapid radiation present in all other cotton clades, after divergence from the Australian cottons.

## Conclusions

Whole chloroplast genome sequencing has been on the rise [68–73], providing an abundance of information both for phylogenetic utility, as well as cytonuclear interactions and accommodation. Here, we report the generation of 6 new *Gossypium* chloroplast genomes, and compare these to 13 other cotton chloroplast genomes to evaluate the evolution of the chloroplast as a whole over the entire genus. The data presented here are congruent with prior chloroplast-based phylogenetic analyses, indicating that, in many cases, sequencing of few chloroplast loci may be just as effective as sequencing the entire molecule. The analyses here also revisit a perhaps underappreciated feature of cotton evolutionary history: the propensity for hybridization and introgression on different time scales and among species whose geographic distance renders the occurrence remarkable. The continued incongruence between the nuclear and chloroplast genomes warrants further exploration through increased nuclear representation. Finally, the sequences presented here represent a valuable resource for cytonuclear coevolution in the genus *Gossypium*, as well as future organelle-based studies.

## Supporting Information

S1 Fig Phylogenetic relationships of the nineteen species of *Gossypium* constructed by maximum likelihood based on the whole chloroplast in its entirety (excluding gaps), with IR junction type listed on the right. Numbers above node are the branch length. (Bayesian tree is similar, and therefore not displayed).
(TIF)

S2 Fig Chronogram showing *Gossypium* phylogeny and divergence time variance around the age estimates with *T*. *cacao* as an outgroup. Consensus tree presenting divergence dates produces by the PhyloBayes analysis of the 78 concatenated chloroplast protein-coding exons dataset using three fossil calibration points (S8 Table), the autocorrelated Lognormal relaxed-clock mode, the site-heterogeneous mixture CAT+GTR substitution model, and soft bound 10%. A geological time scale is shown at the bottom. The arrows represent for three calibration points.
(TIF)

S1 Table General features of other *Gossypium* cp genomes cited in this paper. (DOCX)

S2 Table Genes encoded by *Gossypium* chloroplast genomes. Note: *, ** gene containing a single or two introns, respectively. §, The gene has two copies.
(DOCX)

S3 Table The overall nucleotide distance (coding + non-coding with an IR excluded, excluding indels) among the 19 cotton species. Note: A_1_ = *G*. *herbaceum*, A_1-a_ = *G*. *africanum*, A_2_ = *G*. *arboreum*, AD_1_ = *G*. *hirsutum*, AD_2_ = *G*. *barbadense*, F_1_ = *G*. *longicalyx*, E_1_ = *G*. *stocksii*, E_2_ = *G*. *somalense*, E_3_ = *G*. *areysianum*, E_4_ = *G*. *incanum*, D_1_ = *G*. *thurberi*, D_5_ = *G*. *raimondii*, D_6_ = *G*. *gossypioides*, B_1_ = *G*. *anomalum*, B_3_ = *G*. *capitis-viridis*, C_1_ = *G*. *sturtianum*, C_2_ = *G*. *robinsonii*, G_1_ = *G*. *bickii*, K = *G*. *populifolium*.
(DOCX)

S4 Table The nucleotide distance between 19 *Gossypium* species. Note: The upper triangle shows the number of substitutions in protein-coding exon regions and the lower triangle shows the number of substitutions in non-coding regions. The repeated sequences, naturally, sometimes complicate the alignment process, so we removed an IR region from all chloroplast genomes aligned here. A_1_ = *G*. *herbaceum*, A_1-a_ = *G*. *africanum*, A_2_ = *G*. *arboreum*, AD_1_ = *G*. *hirsutum*, AD_2_ = *G*. *barbadense*, F_1_ = *G*. *longicalyx*, E_1_ = *G*. *stocksii*, E_2_ = *G*. *somalense*, E_3_ = *G*. *areysianum*, E_4_ = *G*. *incanum*, D_1_ = *G*. *thurberi*, D_5_ = *G*. *raimondii*, D_6_ = *G*. *gossypioides*, B_1_ = *G*. *anomalum*, B_3_ = *G*. *capitis-viridis*, C_1_ = *G*. *sturtianum*, C_2_ = *G*. *robinsonii*, G_1_ = *G*. *bickii*, K = *G*. *populifolium*.
(DOCX)

S5 Table Mean nucleotide distances of protein-coding exons and non-coding regions among 19 *Gossypium* species. Note: yellow colors indicate the minimum distances, green colors indicate the maximum distance and NA indicates that there exists overlap sequences between two genes. *clpP* and *ycf3* both contain two introns, while we merged them into one.
(XLSX)

S6 Table Indel length description and Indels data matrix for phylogenetic analysis. Note: Indels were coded as unordered characters with binary states (in the case of simple presence/absence indels) or multistate characters (in the case of indels with variable length but one identical 5’ or 3’ end).
(XLSX)

S7 Table Indels that discriminate *Gossypium* cp genomes. Note: The upper triangle shows the number of indels in protein-coding exon regions and the lower triangle shows the number of indels in non-coding regions. The repeated sequences, naturally, sometimes complicate the alignment process, so we excluded the IR region from the analysis. A_1_ = *G*. *herbaceum*, A_1-a_ = *G*. *africanum*, A_2_ = *G*. *arboreum*, AD_1_ = *G*. *hirsutum*, AD_2_ = *G*. *barbadense*, F_1_ = *G*. *longicalyx*, E_1_ = *G*. *stocksii*, E_2_ = *G*. *somalense*, E_3_ = *G*. *areysianum*, E_4_ = *G*. *incanum*, D_1_ = *G*. *thurberi*, D_5_ = *G*. *raimondii*, D_6_ = *G*. *gossypioides*, B_1_ = *G*. *anomalum*, B_3_ = *G*. *capitis-viridis*, C_1_ = *G*. *sturtianum*, C_2_ = *G*. *robinsonii*, G_1_ = *G*. *bickii*, K = *G*. *populifolium*.
(DOCX)

S8 Table Calibrations with fossil taxonomic information, fossil age and references. (DOCX)

## Abbreviations

*G*: *Gossypium*
tRNAs: transfer RNAs
cpDNA: chloroplast DNA
MYA: million years ago
Cp: chloroplast
Indel: insertion and deletion
PCR: polymerase chain reaction
IR: inverted repeat
SSC region: small single-copy region
LSC region: large single-copy region
ML: Maximum likelihood
