# Supplementary figures and images for: Chloroplast DNA Structural Variation, Phylogeny, and Age of Divergence among Diploid Cotton Species
Source: PLoS One. 2016 Jun 16;11(6):e0157183. doi: 10.1371/journal.pone.0157183 (PMC4911064; doi:10.1371/journal.pone.0157183)

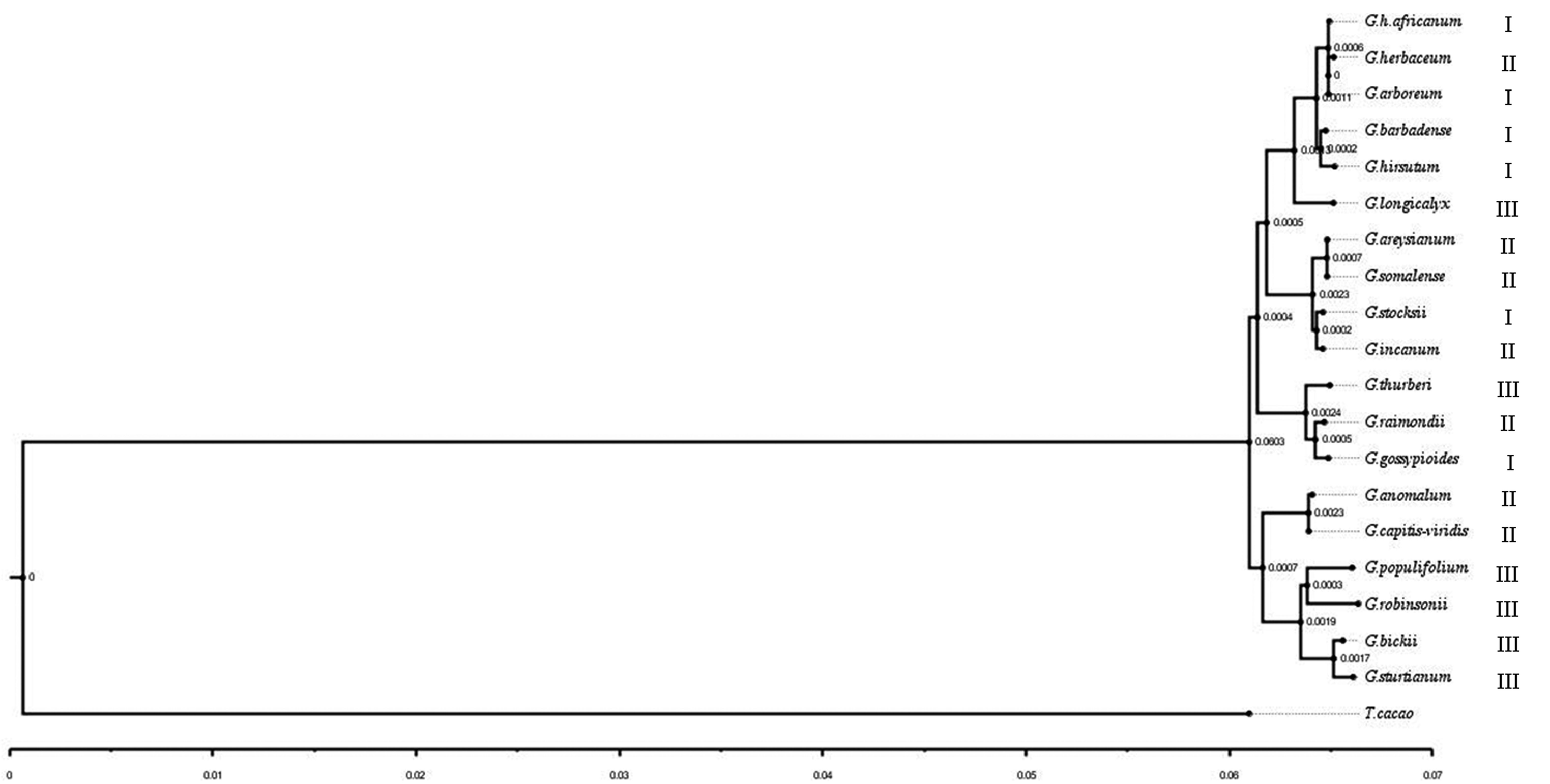

Supplement: S1 Fig — Numbers above node are the branch length. (Bayesian tree is similar, and therefore not displayed). (TIF) [file pone.0157183.s001.tif]

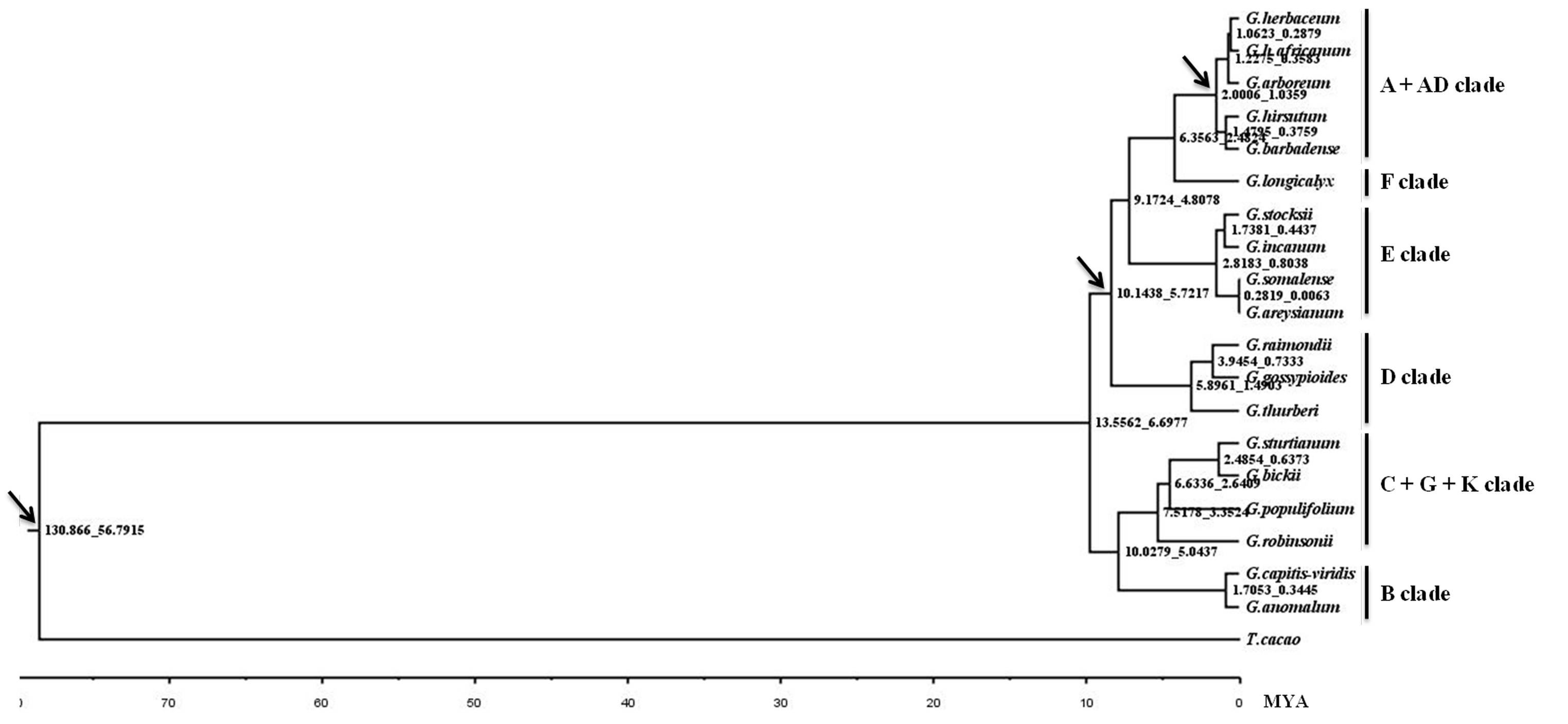

Supplement: S2 Fig — Consensus tree presenting divergence dates produces by the PhyloBayes analysis of the 78 concatenated chloroplast protein-coding exons dataset using three fossil calibration points (S8 Table), the autocorrelated Lognormal relaxed-clock mode, the site-heterogeneous mixture CAT+GTR substitution model, and soft bound 10%. A geological time scale is shown at the bottom. The arrows represent for three calibration points. (TIF) [file pone.0157183.s002.tif]
