# Supplementary material for: Chloroplast DNA Structural Variation, Phylogeny, and Age of Divergence among Diploid Cotton Species
Source: PLoS One. 2016 Jun 16;11(6):e0157183. doi: 10.1371/journal.pone.0157183 (PMC4911064; doi:10.1371/journal.pone.0157183)
Supplement: S1 Table — (DOCX) [file pone.0157183.s003.docx]

**S1 Table. General features of other *Gossypium* cp genomes cited in this paper.**

| Species | Genome type | Genome size (bp) | | | | Coding sequences (bp) | Non-coding sequences (bp) | GenBank accessions | Reference |
| --- | --- | --- | --- | --- | --- | --- | --- | --- | --- |
|  |  | Total | LSC | IR | SSC |  |  |  |  |
| *G. herbaceum* | A**_1_** | 160,264 | 88,737 | 25,628 | 20,271 | 90,633 | 69,631 | GU907100 | - |
| *G. herbaceum* subsp. *africanum* | A_1-a_ | 160,315 | 88,790 | 25,620 | 20,285 | 90,549 | 69,766 | HQ325742 | Xu et al. 2012 |
| *G*. *arboreum* | A_2_ | 160,230 | 88,722 | 25,617 | 20,274 | 90,549 | 69,681 | HQ325740 | Xu et al. 2012 |
| *G*. *anomalum* | B_1_ | 159,505 | 88,102 | 25,602 | 20,199 | 90,552 | 68,953 | JF317351 | - |
| *G*. *sturtianum* | C_1_ | 159,627 | 88,251 | 25,580 | 20,216 | 90,558 | 69,069 | JF317356 | - |
| *G. thurberi* | D**_1_** | 160,140 | 88,709 | 25,605 | 20,221 | 90,633 | 69,507 | JF317353 | - |
| *G*. *raimondii* | D_5_ | 160,161 | 88,654 | 25,651 | 20,205 | 90,558 | 69,603 | HQ325744 | Xu et al. 2012 |
| *G*. *gossypioides* | D_6_ | 159,959 | 88,777 | 25,589 | 20,004 | 90,561 | 69,398 | HQ901195 | Xu et al. 2012 |
| *G*. *stocksii* | E_1_ | 159,039 | 87,886 | 25,487 | 20,179 | 90,525 | 68,514 | JF317355 | - |
| *G*. *longicalyx* | F_1_ | 160,241 | 88,667 | 25,648 | 20,278 | 90,561 | 69,680 | JF317354 | - |
| *G*. *bickii* | G_1_ | 159,422 | 88,073 | 25,583 | 20,183 | 90,546 | 68,876 | JF317352 | - |
| *G. hirsutum* | AD_1_ | 160,301 | 88,817 | 25,602 | 20,280 | 90,555 | 69,746 | DQ345959 | Lee et al. 2006 |
| *G. barbadense* | AD_2_ | 160,302 | 88,849 | 25,593 | 20,267 | 90,555 | 69,747 | HQ901199 | Xu et al. 2012 |
