# Supplementary material for: Chloroplast DNA Structural Variation, Phylogeny, and Age of Divergence among Diploid Cotton Species
Source: PLoS One. 2016 Jun 16;11(6):e0157183. doi: 10.1371/journal.pone.0157183 (PMC4911064; doi:10.1371/journal.pone.0157183)
Supplement: S2 Table — Note: *, ** gene containing a single or two introns, respectively. §, The gene has two copies. (DOCX) [file pone.0157183.s004.docx]

**S2 Table. Genes encoded by *Gossypium* chloroplast genomes.**

| **Category** | | **Genes** | |
| --- | --- | --- | --- |
| **Photosynthesis related genes** | |  |  |
|  | RuBisCO large subunit: | *rbcL* |  |
|  | Photosystem I genes: | *psaA*, *psaB*, *psaC*, *psaI*, *psaJ* |  |
|  | Assembly/stability of photosystem I: | *ycf3*, *ycf4* |  |
|  | Photosystem II genes: | *psbA*, *psbB*, *psbC*, *psbD*, *psbE*, *psbF*, *psbH*, *psbI*, *psbJ*, *psbK*, *psbL*, *psbM*, *psbN*, *psbT*, *psbZ* | |
|  | Cytochrome b/f complex genes: | *petA*, *petB*, *petD*, *petG*, *petL*, *petN* |  |
|  | *c-*type cytochrome: | *ccsA* |  |
|  | ATP synthase genes: | *atpA*, *atpB*, *atpE*, *atpF*, *atpH*, *atpI* |  |
|  | NADH dehydrogenase genes: | *ndhA*, *ndhB*§, *ndhC*, *ndhD*, *ndhE*, *ndhF*, *ndhG*, *ndhH*, *ndhI*, *ndhJ*, *ndhK* | |
| **Transcription and translation related genes** | |  |  |
|  | RNA polymerase and related genes: | *rpoA*, *rpoB*, *rpoC1**, *rpoC2* |  |
|  | Ribosomal protein genes: | *rps2*, *rps3*, *rps4*, *rps7*§, *rps8*, *rps11*, *rps12***§, *rps14*, *rps15*, *rps16**, *rps18*, | |
|  |  | *rps19*, *rpl2**§, *rpl14*, *rpl16**, *rpl20*, *rpl22*, *rpl23*§, *rpl32*, *rpl33*, *rpl36* | |
| **RNA genes** | |  |  |
|  | Ribosomal RNA genes: | *rrn23*§, *rrn16*§, *rrn5*§, *rrn4.5*§ |  |
|  | Transfer RNA genes: | *trnA(UGC)**§, *trnC(GCA)*, *trnD(GUC)*, *trnE(UUC)*, *trnF(GAA)*, *trnG(GCC)*, *trnG(UCC)**, *trnH(GUG)*, *trnI(CAU)*§, *trnI(GAU)**§, *trnK(UUU)**, *trnL(CAA)*§, *trnL(UAA)**, *trnL(UAG)*, *trnfM(CAU)*, *trnM(CAU)*, *trnN(GUU)*§, *trnP(UGG)*, *trnQ(UUG)*, *trnR(ACG)*§, *trnR(UCU)*, *trnS(GCU)*, *trnS(GGA)*, *trnS(UGA)*, *trnT(GGU)*, *trnT(UGU)*, *trnV(GAC)*§, *trnV(UAC)**,  *trnW(CCA)*,  *trnY(GUA)* | |
|  |  |  |  |
|  |  |  |  |
|  |  |  |  |
|  |  |  |  |
| **Others** | |  |  |
|  | Maturase | *matK* |  |
|  | Acetyl-CoA carboxylase subunit: | *accD* |  |
|  | ATP-dependent protease subunit: | *clpP*** |  |
|  | Inorganic carbon uptake: | *cemA* |  |
|  | Conserved reading frames (*ycf*s): | *ycf1*, *ycf2*§ |  |

Note: *, ** gene containing a single or two introns, respectively. §, The gene has two copies.
