# Supplementary material for: Chloroplast DNA Structural Variation, Phylogeny, and Age of Divergence among Diploid Cotton Species
Source: PLoS One. 2016 Jun 16;11(6):e0157183. doi: 10.1371/journal.pone.0157183 (PMC4911064; doi:10.1371/journal.pone.0157183)
Supplement: S3 Table — Note: A1 = G. herbaceum, A1-a = G. africanum, A2 = G. arboreum, AD1 = G. hirsutum, AD2 = G. barbadense, F1 = G. longicalyx, E1 = G. stocksii, E2 = G. somalense, E3 = G. areysianum, E4 = G. incanum, D1 = G. thurberi, D5 = G. raimondii, D6 = G. gossypioides, B1 = G. anomalum, B3 = G. capitis-viridis, C1 = G. sturtianum, C2 = G. robinsonii, G1 = G. bickii, K = G. populifolium. (DOCX) [file pone.0157183.s005.docx]

**S3 Table. The overall nucleotide distance (coding + non-coding with an IR excluded, excluding indels) among the 19 cotton species.**

|  | A_1_ | A_1-a_ | A_2_ | AD_1_ | AD_2_ | F_1_ | E_1_ | E_2_ | E_3_ | E_4_ | D_1_ | D_5_ | D_6_ | B_1_ | B_3_ | C_1_ | C_2_ | G_1_ |
| --- | --- | --- | --- | --- | --- | --- | --- | --- | --- | --- | --- | --- | --- | --- | --- | --- | --- | --- |
| A_1-a_ | 0.000334 |  |  |  |  |  |  |  |  |  |  |  |  |  |  |  |  |  |
| A_2_ | 0.000275 | 0.000074 |  |  |  |  |  |  |  |  |  |  |  |  |  |  |  |  |
| AD_1_ | 0.001840 | 0.001654 | 0.001624 |  |  |  |  |  |  |  |  |  |  |  |  |  |  |  |
| AD_2_ | 0.001437 | 0.001251 | 0.001221 | 0.001087 |  |  |  |  |  |  |  |  |  |  |  |  |  |  |
| F_1_ | 0.003952 | 0.003757 | 0.003720 | 0.003953 | 0.003593 |  |  |  |  |  |  |  |  |  |  |  |  |  |
| E_1_ | 0.005599 | 0.005448 | 0.005417 | 0.005683 | 0.005495 | 0.005981 |  |  |  |  |  |  |  |  |  |  |  |  |
| E_2_ | 0.005930 | 0.005741 | 0.005711 | 0.006067 | 0.005841 | 0.006340 | 0.001320 |  |  |  |  |  |  |  |  |  |  |  |
| E_3_ | 0.005959 | 0.005771 | 0.005740 | 0.006112 | 0.005870 | 0.006384 | 0.001350 | 0.000075 |  |  |  |  |  |  |  |  |  |  |
| E_4_ | 0.005602 | 0.005413 | 0.005383 | 0.005724 | 0.005475 | 0.006014 | 0.000668 | 0.001237 | 0.001267 |  |  |  |  |  |  |  |  |  |
| D_1_ | 0.006627 | 0.006484 | 0.006469 | 0.006742 | 0.006501 | 0.006895 | 0.006570 | 0.006958 | 0.007018 | 0.006694 |  |  |  |  |  |  |  |  |
| D_5_ | 0.006530 | 0.006364 | 0.006341 | 0.006781 | 0.006441 | 0.006760 | 0.006623 | 0.006860 | 0.006934 | 0.006580 | 0.002356 |  |  |  |  |  |  |  |
| D_6_ | 0.006863 | 0.006712 | 0.006674 | 0.007024 | 0.006775 | 0.007178 | 0.006815 | 0.007037 | 0.007088 | 0.006870 | 0.002516 | 0.001283 |  |  |  |  |  |  |
| B_1_ | 0.006551 | 0.006377 | 0.006346 | 0.006765 | 0.006515 | 0.007095 | 0.006534 | 0.006845 | 0.006875 | 0.006576 | 0.006991 | 0.007074 | 0.007320 |  |  |  |  |  |
| B_3_ | 0.006558 | 0.006354 | 0.006324 | 0.006719 | 0.006469 | 0.007042 | 0.006413 | 0.006633 | 0.006678 | 0.006348 | 0.006968 | 0.006938 | 0.007162 | 0.000284 |  |  |  |  |
| C_1_ | 0.008666 | 0.008537 | 0.008499 | 0.008933 | 0.008614 | 0.008902 | 0.008223 | 0.008740 | 0.008770 | 0.008355 | 0.009173 | 0.009287 | 0.009612 | 0.007737 | 0.007632 |  |  |  |
| C_2_ | 0.008854 | 0.008672 | 0.008634 | 0.009212 | 0.008856 | 0.009334 | 0.008554 | 0.008870 | 0.008899 | 0.008487 | 0.009083 | 0.009009 | 0.009355 | 0.007491 | 0.007248 | 0.006305 |  |  |
| G_1_ | 0.008347 | 0.008203 | 0.008165 | 0.008561 | 0.008227 | 0.008659 | 0.007743 | 0.008239 | 0.008269 | 0.007830 | 0.008839 | 0.008893 | 0.009195 | 0.007076 | 0.006970 | 0.001625 | 0.005537 |  |
| K | 0.008708 | 0.008526 | 0.008488 | 0.009105 | 0.008709 | 0.009158 | 0.008353 | 0.008739 | 0.008754 | 0.008309 | 0.008998 | 0.008566 | 0.008859 | 0.007242 | 0.007014 | 0.005651 | 0.005205 | 0.004867 |

Note: A_1_ = *G. herbaceum*, A_1-a_ = *G. africanum*, A_2_ = *G. arboreum*, AD_1_ = *G. hirsutum*, AD_2_ = *G. barbadense*, F_1_ = *G. longicalyx* , E_1_ = *G. stocksii*, E_2_ = *G. somalense* , E_3_ = *G. areysianum*, E_4_ = *G. incanum*, D_1_ = *G. thurberi*, D_5_ = *G. raimondii*, D_6_ = *G. gossypioides*, B_1_ = *G. anomalum*, B_3_ = *G. capitis-viridis*, C_1_ = *G. sturtianum*, C_2_ = *G. robinsonii*, G_1_ = *G. bickii*, K= *G. populifolium*.
