# Supplementary material for: Chloroplast DNA Structural Variation, Phylogeny, and Age of Divergence among Diploid Cotton Species
Source: PLoS One. 2016 Jun 16;11(6):e0157183. doi: 10.1371/journal.pone.0157183 (PMC4911064; doi:10.1371/journal.pone.0157183)
Supplement: S4 Table — Note: The upper triangle shows the number of substitutions in protein-coding exon regions and the lower triangle shows the number of substitutions in non-coding regions. The repeated sequences, naturally, sometimes complicate the alignment process, so we removed an IR region from all chloroplast genomes aligned here. A1 = G. herbaceum, A1-a = G. africanum, A2 = G. arboreum, AD1 = G. hirsutum, AD2 = G. barbadense, F1 = G. longicalyx, E1 = G. stocksii, E2 = G. somalense, E3 = G. areysianum, E4 = G. incanum, D1 = G. thurberi, D5 = G. raimondii, D6 = G. gossypioides, B1 = G. anomalum, B3 = G. capitis-viridis, C1 = G. sturtianum, C2 = G. robinsonii, G1 = G. bickii, K = G. populifolium. (DOCX) [file pone.0157183.s006.docx]

**S4 Table. The nucleotide distance between 19 *Gossypium* species.**

| Species | A_1_ | A_1-a_ | A_2_ | AD_1_ | AD_2_ | B_1_ | B_3_ | C_1_ | C_2_ | D_1_ | D_5_ | D_6_ | E~~1~~ | E_2_ | E_3_ | E_4_ | F_1_ | G_1_ | K |
| --- | --- | --- | --- | --- | --- | --- | --- | --- | --- | --- | --- | --- | --- | --- | --- | --- | --- | --- | --- |
| A_1_ |  | 0.000383 | 0.000339 | 0.000737 | 0.000737 | 0.003907 | 0.003863 | 0.004836 | 0.005087 | 0.00376 | 0.003863 | 0.003819 | 0.00345 | 0.00376 | 0.003774 | 0.00373 | 0.002226 | 0.004644 | 0.004925 |
| A_1-a_ | 0.000222 |  | 8.85E-05 | 0.000546 | 0.000546 | 0.003745 | 0.003686 | 0.004718 | 0.00491 | 0.003627 | 0.00373 | 0.003686 | 0.003347 | 0.003568 | 0.003583 | 0.003524 | 0.00202 | 0.004497 | 0.004762 |
| A_2_ | 0.000163 | 5.83E-05 |  | 0.000487 | 0.000487 | 0.003701 | 0.003627 | 0.004674 | 0.004851 | 0.003612 | 0.003701 | 0.003657 | 0.003303 | 0.003509 | 0.003524 | 0.00348 | 0.001976 | 0.004453 | 0.004703 |
| AD_1_ | 0.002228 | 0.002182 | 0.00217 |  | 0.000354 | 0.00373 | 0.003686 | 0.004703 | 0.004925 | 0.003657 | 0.003774 | 0.00376 | 0.003376 | 0.003642 | 0.003657 | 0.003583 | 0.00202 | 0.004482 | 0.004792 |
| AD_2_ | 0.001552 | 0.001412 | 0.001447 | 0.001423 |  | 0.003878 | 0.003833 | 0.004659 | 0.004807 | 0.00348 | 0.003598 | 0.003583 | 0.003362 | 0.003657 | 0.003642 | 0.003598 | 0.001814 | 0.004438 | 0.004748 |
| B_1_ | 0.006475 | 0.006591 | 0.00658 | 0.007735 | 0.006988 |  | 0.000251 | 0.003229 | 0.004114 | 0.003715 | 0.00376 | 0.003774 | 0.003745 | 0.00376 | 0.003966 | 0.003907 | 0.003819 | 0.003627 | 0.003907 |
| B_3_ | 0.006918 | 0.006941 | 0.006941 | 0.007945 | 0.007221 | 0.00028 |  | 0.003833 | 0.003922 | 0.003657 | 0.003583 | 0.003598 | 0.003701 | 0.003774 | 0.003789 | 0.003715 | 0.003745 | 0.003583 | 0.003715 |
| C_1_ | 0.008971 | 0.009158 | 0.009111 | 0.009928 | 0.008866 | 0.008621 | 0.007781 |  | 0.002742 | 0.004866 | 0.004954 | 0.004939 | 0.004748 | 0.005087 | 0.005101 | 0.005013 | 0.004821 | 0.000722 | 0.002905 |
| C_2_ | 0.008586 | 0.008703 | 0.008773 | 0.009869 | 0.008995 | 0.007886 | 0.007641 | 0.006801 |  | 0.004895 | 0.004276 | 0.004851 | 0.004954 | 0.004187 | 0.005042 | 0.004998 | 0.003804 | 0.00314 | 0.003082 |
| D_1_ | 0.006591 | 0.006521 | 0.006463 | 0.007105 | 0.006708 | 0.00693 | 0.006813 | 0.008901 | 0.009053 |  | 0.001327 | 0.001283 | 0.003671 | 0.003878 | 0.003892 | 0.003848 | 0.003627 | 0.004615 | 0.004748 |
| D_5_ | 0.006801 | 0.006953 | 0.00693 | 0.007898 | 0.006965 | 0.00742 | 0.007233 | 0.009414 | 0.010254 | 0.002707 |  | 0.000899 | 0.00373 | 0.003804 | 0.003819 | 0.003774 | 0.00376 | 0.004703 | 0.004836 |
| D_6_ | 0.007618 | 0.007711 | 0.007676 | 0.008481 | 0.007513 | 0.007688 | 0.007501 | 0.009951 | 0.010383 | 0.002987 | 0.001353 |  | 0.00376 | 0.003789 | 0.003804 | 0.003819 | 0.003745 | 0.004689 | 0.004792 |
| E_1_ | 0.00546 | 0.00553 | 0.005483 | 0.006545 | 0.005985 | 0.006801 | 0.006498 | 0.009286 | 0.009169 | 0.007046 | 0.007548 | 0.007746 |  | 0.000649 | 0.000663 | 0.000383 | 0.003244 | 0.003937 | 0.004939 |
| E_2_ | 0.006323 | 0.006521 | 0.006475 | 0.007245 | 0.00651 | 0.007641 | 0.007128 | 0.010009 | 0.010313 | 0.007898 | 0.008248 | 0.00826 | 0.001657 |  | 1.47E-05 | 0.00059 | 0.003509 | 0.004836 | 0.004969 |
| E_3_ | 0.006323 | 0.006521 | 0.006475 | 0.007373 | 0.006521 | 0.00756 | 0.007233 | 0.010033 | 0.010103 | 0.007851 | 0.008376 | 0.008341 | 0.001505 | 0.000117 |  | 0.000605 | 0.003524 | 0.004851 | 0.004983 |
| E_4_ | 0.005518 | 0.005705 | 0.005658 | 0.006498 | 0.005833 | 0.006661 | 0.006346 | 0.009251 | 0.00882 | 0.007175 | 0.007338 | 0.007828 | 0.000677 | 0.001493 | 0.001482 |  | 0.003435 | 0.004762 | 0.005013 |
| F_1_ | 0.004095 | 0.00413 | 0.004095 | 0.004701 | 0.00406 | 0.007956 | 0.007886 | 0.009683 | 0.011141 | 0.007315 | 0.007525 | 0.008213 | 0.006591 | 0.007256 | 0.007245 | 0.006533 |  | 0.0046 | 0.004939 |
| G_1_ | 0.008283 | 0.008516 | 0.008423 | 0.009414 | 0.008423 | 0.007653 | 0.00756 | 0.00196 | 0.005098 | 0.008808 | 0.009065 | 0.009659 | 0.009461 | 0.009379 | 0.009449 | 0.008855 | 0.009356 |  | 0.002683 |
| K | 0.009158 | 0.008971 | 0.008971 | 0.010126 | 0.007606 | 0.010581 | 0.010429 | 0.006696 | 0.005611 | 0.009519 | 0.009134 | 0.010114 | 0.008563 | 0.009624 | 0.009671 | 0.008598 | 0.010173 | 0.005425 |  |

Note: The upper triangle shows the number of substitutions in protein-coding exon regions and the lower triangle shows the number of substitutions in non-coding regions. The repeated sequences, naturally, sometimes complicate the alignment process, so we removed an IR region from all chloroplast genomes aligned here. A_1_ = *G. herbaceum*, A_1-a_ = *G. africanum*, A_2_ = *G. arboreum*, AD_1_ = *G. hirsutum*, AD_2_ = *G. barbadense*, F_1_ = *G. longicalyx* , E_1_ = *G. stocksii*, E_2_ = *G. somalense* , E_3_ = *G. areysianum*, E_4_ = *G. incanum*, D_1_ = *G. thurberi*, D_5_ = *G. raimondii*, D_6_ = *G. gossypioides*, B_1_ = *G. anomalum*, B_3_ = *G. capitis-viridis*, C_1_ = *G. sturtianum*, C_2_ = *G. robinsonii*, G_1_ = *G. bickii*, K= *G. populifolium*.
