# Supplementary material for: Chloroplast DNA Structural Variation, Phylogeny, and Age of Divergence among Diploid Cotton Species
Source: PLoS One. 2016 Jun 16;11(6):e0157183. doi: 10.1371/journal.pone.0157183 (PMC4911064; doi:10.1371/journal.pone.0157183)
Supplement: S7 Table — Note: The upper triangle shows the number of indels in protein-coding exon regions and the lower triangle shows the number of indels in non-coding regions. The repeated sequences, naturally, sometimes complicate the alignment process, so we excluded the IR region from the analysis. A1 = G. herbaceum, A1-a = G. africanum, A2 = G. arboreum, AD1 = G. hirsutum, AD2 = G. barbadense, F1 = G. longicalyx, E1 = G. stocksii, E2 = G. somalense, E3 = G. areysianum, E4 = G. incanum, D1 = G. thurberi, D5 = G. raimondii, D6 = G. gossypioides, B1 = G. anomalum, B3 = G. capitis-viridis, C1 = G. sturtianum, C2 = G. robinsonii, G1 = G. bickii, K = G. populifolium. (DOCX) [file pone.0157183.s009.docx]

**S7 Table. Indels that discriminate *Gossypium* cp genomes.**

| Species | A1 | A1-a | A2 | AD1 | AD2 | B1 | B3 | C1 | C2 | D1 | D5 | D6 | E1 | E2 | E3 | E4 | F1 | G1 | K |
| --- | --- | --- | --- | --- | --- | --- | --- | --- | --- | --- | --- | --- | --- | --- | --- | --- | --- | --- | --- |
| A1 |  | 5 | 5 | 10 | 14 | 8 | 14 | 4 | 11 | 5 | 8 | 9 | 8 | 9 | 9 | 14 | 4 | 5 | 12 |
| A1-a | 22 |  | 0 | 5 | 9 | 8 | 8 | 3 | 5 | 10 | 3 | 4 | 7 | 3 | 4 | 6 | 5 | 5 | 7 |
| A2 | 14 | 13 |  | 5 | 9 | 8 | 8 | 3 | 5 | 10 | 3 | 4 | 7 | 3 | 4 | 6 | 5 | 5 | 7 |
| AD1 | 108 | 117 | 113 |  | 4 | 10 | 10 | 4 | 6 | 11 | 4 | 5 | 10 | 6 | 7 | 9 | 6 | 6 | 8 |
| AD2 | 103 | 103 | 96 | 75 |  | 17 | 11 | 14 | 9 | 15 | 8 | 9 | 18 | 7 | 7 | 12 | 14 | 15 | 12 |
| B1 | 264 | 277 | 270 | 278 | 251 |  | 2 | 6 | 10 | 8 | 8 | 9 | 11 | 7 | 8 | 10 | 9 | 10 | 17 |
| B3 | 254 | 272 | 265 | 272 | 255 | 24 |  | 8 | 10 | 16 | 8 | 9 | 11 | 7 | 8 | 10 | 10 | 10 | 13 |
| C1 | 222 | 295 | 290 | 310 | 284 | 260 | 253 |  | 2 | 3 | 0 | 1 | 8 | 4 | 5 | 7 | 4 | 2 | 10 |
| C2 | 284 | 300 | 291 | 306 | 295 | 267 | 259 | 215 |  | 10 | 2 | 3 | 10 | 6 | 7 | 9 | 6 | 4 | 7 |
| D1 | 245 | 237 | 231 | 237 | 234 | 227 | 211 | 219 | 239 |  | 7 | 8 | 11 | 12 | 12 | 17 | 3 | 4 | 11 |
| D5 | 249 | 259 | 253 | 257 | 252 | 233 | 221 | 241 | 257 | 95 |  | 1 | 8 | 4 | 5 | 7 | 4 | 2 | 4 |
| D6 | 247 | 259 | 253 | 257 | 252 | 252 | 240 | 240 | 258 | 90 | 68 |  | 9 | 5 | 6 | 8 | 5 | 3 | 5 |
| E1 | 294 | 316 | 309 | 311 | 280 | 284 | 273 | 308 | 311 | 255 | 266 | 274 |  | 4 | 5 | 3 | 10 | 10 | 17 |
| E2 | 243 | 263 | 256 | 271 | 247 | 247 | 234 | 259 | 266 | 197 | 206 | 215 | 148 |  | 1 | 3 | 6 | 6 | 9 |
| E3 | 245 | 264 | 257 | 272 | 250 | 251 | 239 | 261 | 267 | 200 | 204 | 216 | 150 | 4 |  | 4 | 7 | 7 | 9 |
| E4 | 274 | 293 | 287 | 289 | 268 | 261 | 252 | 293 | 294 | 233 | 246 | 251 | 84 | 121 | 123 |  | 9 | 9 | 13 |
| F1 | 167 | 173 | 170 | 193 | 169 | 245 | 238 | 266 | 272 | 216 | 222 | 219 | 280 | 233 | 234 | 269 |  | 6 | 12 |
| G1 | 297 | 305 | 301 | 319 | 299 | 254 | 243 | 88 | 205 | 228 | 238 | 236 | 307 | 260 | 260 | 289 | 268 |  | 11 |
| K | 280 | 285 | 278 | 287 | 273 | 252 | 248 | 192 | 191 | 218 | 225 | 234 | 274 | 238 | 238 | 264 | 238 | 187 |  |

Note: The upper triangle shows the number of indels in protein-coding exon regions and the lower triangle shows the number of indels in non-coding regions. The repeated sequences, naturally, sometimes complicate the alignment process, so we excluded the IR region from the analysis. A_1_ = *G. herbaceum*, A_1-a_ = *G. africanum*, A_2_ = *G. arboreum*, AD_1_ = *G. hirsutum*, AD_2_ = *G. barbadense*, F_1_ = *G. longicalyx* , E_1_ = *G. stocksii*, E_2_ = *G. somalense* , E_3_ = *G. areysianum*, E_4_ = *G. incanum*, D_1_ = *G. thurberi*, D_5_ = *G. raimondii*, D_6_ = *G. gossypioides*, B_1_ = *G. anomalum*, B_3_ = *G. capitis-viridis*, C_1_ = *G. sturtianum*, C_2_ = *G. robinsonii*, G_1_ = *G. bickii*, K= *G. populifolium*.
