# Supplementary material for: Chloroplast DNA Structural Variation, Phylogeny, and Age of Divergence among Diploid Cotton Species
Source: PLoS One. 2016 Jun 16;11(6):e0157183. doi: 10.1371/journal.pone.0157183 (PMC4911064; doi:10.1371/journal.pone.0157183)
Supplement: S8 Table — (DOCX) [file pone.0157183.s010.docx]

**S8 Table. Calibrations with fossil taxonomic information, fossil age and references.**

| Calibrations | Node assigned | Minimum age (MYA) | Maximum age (MYA) | References |
| --- | --- | --- | --- | --- |
| Calibration 1 | *Theobroma*-*Gossypium* | 60 | +∞ | 49 |
| Calibration 2 | *G. arboreum*-*G. raimondii* | 5 | 10 | 51 |
| Calibration 3 | *G. arboreum*-*G. hirsutum* | 1 | 2 | 50 |
